# Supplementary material for: Are carbon emissions trading and green financial instruments synergistic? -Comprehensive quantitative research based on content analysis
Source: PLoS One. 2024 Mar 7;19(3):e0298601. doi: 10.1371/journal.pone.0298601 (PMC10919601; doi:10.1371/journal.pone.0298601)
Supplement: S1 Data — (ZIP) [file pone.0298601.s001.zip › Data/地级市Z_F工作报告.zip!/╒π╜¡/─■▓¿╩╨/└┤╫╘╣1⁄2╓┌║┼í╛┬φ┐╦╩2╛▌═°í┐_2016.docx]

各位代表：

　　现在，我代表市人民政府向大会报告工作，请予审议，并请市政协委员和其他列席人员提出意见。

　　一、2015年主要工作和“十二五”发展成就

　　2015年是不平凡的一年。我们面对错综复杂的宏观环境和繁重艰巨的改革发展稳定任务，在市委的坚强领导下，认真落实党的十八大和十八届三中、四中、五中全会精神，深入贯彻习近平总书记系列重要讲话精神，紧紧围绕省委“八八战略”和“两富”“两美”浙江建设，主动适应经济发展新常态，全面实施市委“六个加快”和“双驱动四治理”战略决策，扎实推进经济社会转型发展三年行动计划，较好地完成了市十四届人大五次会议确定的目标任务。实现地区生产总值8011.5亿元，增长8%；完成财政总收入2072.7亿元、增长11.4%，其中一般公共预算收入1006.4亿元、增长8.2%。

　　一年来，我们主要抓了五个方面的工作：

　　（一）精准施策，千方百计稳定增长。针对内外市场需求疲软、大宗商品价格下降、经济下行压力增大等情况，加强经济运行监测分析，强化“双底线”管理，打好稳增促调组合拳。推动工业和外贸稳定增长，规上工业增加值增长3.8%，工业技改投资增长35.4%，外贸进出口连续三年保持1000亿美元以上。抢抓国家实施“中国制造2025”“互联网+”等战略机遇，培育新产业、新业态、新模式，装备制造业、医药制造业增加值分别增长5.9%和10.1%，网络零售额增长59.7%，跨境电子商务试点进出口总额达到81.4亿元。加快现代服务业发展，全国首家专业航运保险公司开业运营，直接融资总额达到1371.3亿元，宁波现代国际物流园区成为全国现代港口物流品牌示范区，老外滩升级为中国著名商业街。社会消费品零售总额增长12%，商品房成交面积增长38.7%。发展休闲农业、都市农业，农业生产保持稳定，成功创建国家现代农业示范区，渔山列岛海域成为国家级海洋牧场示范区。

　　（二）破难攻坚，扎实推进重点工作。深入实施经济社会转型发展三年行动计划，加强协调服务和要素保障，新开工重大项目261个、续建420个、建成155个。扩大有效投资，完成固定资产投资4506.6亿元，增长13%。轨道交通2号线一期、铁路宁波站交通枢纽等项目建成投用，吉利动力总成中国制造中心、大榭东华能源一期、中海油三期等项目基本建成，宁波新能源汽车、康龙化成生命科技、康达医疗等产业园开工建设，上海大众宁波基地扩建等项目加快推进。积极参与“一带一路”、长江经济带建设，港口经济圈建设纳入长三角城市群规划，成功获批中国（宁波）跨境电子商务综合试验区和国际邮件互换局（交换站）。推进现代化枢纽港建设，大宗商品和航运交易发展迅速，“海上丝路宁波出口集装箱运价指数”登陆波罗的海交易所，宁波舟山港集装箱吞吐量达到2063万标箱。成功举办首届中国—中东欧国家投资贸易博览会、第十七届浙洽会、中国航海日和中国网博会等重大活动。

　　（三）改革创新，切实增强发展活力。加快国际贸易“单一窗口”和口岸大通关建设，实施区域通关一体化和“区区联动”试点，深化全国保险创新综合示范区建设，获批全国普惠金融综合示范区，推广政府与社会资本合作模式，推进国企“瘦身强体”，实现建设项目排污权交易全覆盖。开展国家新型城镇化综合试点，实施城市总体规划、土地利用总体规划、环境功能区划“三规融合”，推进户籍制度改革试点、不动产登记改革和农村产权制度改革，国有林场改革顺利完成。加快政府职能转变，完善“四张清单一张网”，并向乡镇（街道）全覆盖。深化综合行政执法体制改革，完善社会信用体系，实施政府常务会议集体学法制度，加大正风肃纪力度。加快新材料科技城、国际海洋生态科技城建设，引进国际材料基因工程研究院、海洋研究院等创新机构，培育特色小镇和众创空间，电商经济创新园区开园运营，宁波杭州湾新区、梅山国际物流产业集聚区、中意宁波生态园加快发展。优化创业创新环境，设立“四大产业基金”，出台人才新政，实施“五证合一、一照一码”改革和简易注销改革试点，成为国家知识产权区域布局试点城市。发明专利授权量5412件，新增创新型初创企业1848家。

　　（四）统筹协调，加快提升城乡品质。完善大交通体系，加快三门湾大桥、杭州湾大桥杭甬高速连接线建设，栎社国际机场三期扩建、穿山港铁路支线和宁波至奉化城际铁路开工建设，铁路货运北环线全线通车，宁海通用机场顺利获批。优化城乡基础设施，新江桥、夏禹路建成通车，主干道快速化改造稳步实施，水库群联网联调和“两江同治6+1”工程启动建设，五大应急泵站建成投用。实施“提升城乡品质，建设美丽宁波”行动计划，扎实推进“三治理一提高”行动，开展“海绵城市”、地下综合管廊城市和垃圾分类试点，垃圾焚烧项目建设有序推进，乡镇污水处理设施实现全覆盖，农村生活污水治理覆盖率稳步提高。“三江六岸”品质提升步伐加快，中山路整治提升、“两路两侧”整治和城市门户区、高速出入口升级改造全面推进。大力改善生态环境，支持四明山区域生态发展，整治重污染行业，实施“黄标车”全面禁行，淘汰改造高污染燃料锅炉，镇海电厂搬迁有序推进，渔场修复振兴“一打三整治”行动成效明显。节能减排目标全面完成。

　　（五）改善民生，保持社会和谐稳定。十方面民生实事圆满完成。城镇新增就业17.8万人，城乡居民收入分别达到4.8万元和2.6万元，分别增长8.4%和9%。推进社保制度统一并轨，提高低保和养老金标准，扩大医疗救助范围，医保参保人员大病保险制度实现全覆盖。实施持证残疾人免费乘坐市区公共汽车、地铁政策。以成片危旧住宅区为重点的棚户区改造有序开展，提供保障性安居工程3.2万套。推进文化繁荣发展，出台历史文化名城保护规划，加快市图书馆新馆建设，组建宁波交响乐团，新闻客户端“甬派”上线运营，它山堰成为世界灌溉工程遗产，文明城市创建深入推进。提升发展社会事业，宁波大学成为省首批重点建设高校，工程学院新校区基本建成，宁波杭州湾汽车学院、李惠利东部医院建成投用，全国首家云医院上线运营，家庭医生制签约服务全面实施，成功举办第十七届市运会和首届宁波国际马拉松赛。创新社会治理，健全立体化社会治安防控体系，严厉打击违法犯罪活动，完善信访维稳和矛盾化解机制，构建食品药品安全“两网五体系”，推进重点领域“打非治违”，安全生产事故和死亡人数继续下降。

　　各位代表！2015年是“十二五”规划的收官之年，我们为“十二五”发展划上了圆满的句号。五年来，我们深入贯彻落实省委、省政府和市委的决策部署，认真执行市人大及其常委会的决议决定，全市经济社会发展上了一个新台阶。

　　五年来，我们坚持扩总量、促增量、提质量，综合实力稳步提高。经济平稳健康发展，地区生产总值年均增长8.3%，人均地区生产总值超过1.6万美元。财政总收入、一般公共预算收入年均分别增长12.1%和13.6%。国际强港建设稳步推进，宁波舟山港集装箱吞吐量全球排名由第六位上升到第四位，集装箱海铁联运量年均增长44%。人才规模不断扩大，拥有“千人计划”人才271名，人才总量达到187.2万人。创新能力稳步提高，获批全国首批质量强市示范城市、国家知识产权示范城市，创新能力跃居全国第八位。城市品牌稳步提升，荣获全国文明城市“四连冠”和中国小康社会建设示范奖，成为世界文化遗产城市和“东亚文化之都”，基本公共服务满意度居全国38个大中城市第二位，政府透明度连续多年位居副省级城市和计划单列市首位，多次荣获“中国最具投资吸引力城市”“中国最具幸福感城市”称号。

　　五年来，我们坚持强创新、调结构、增后劲，转型发展步伐加快。深化国家创新型城市试点，发明专利授权量年均增长35%，研究与试验发展经费支出占地区生产总值比重从1.6%提高到2.4%，高新技术企业从662家增加到1519家。扩大有效投资，完成固定资产投资1.7万亿元，建成铁路158公里、高速公路111公里；轨道交通从无到有，建成运营里程达到49公里。建设工业强市，实施“四换三名”工程，规上工业总产值达到1.4万亿元，高新技术产业、装备制造业增加值占规上工业增加值比重分别达到37%和45.4%。培育现代服务业，服务业增加值占地区生产总值比重提高到47.4%，社会消费品零售总额年均增长14.5%，金融机构本外币存贷款余额年均分别增长10.6%和10.8%，年旅游总收入达到1233亿元。发展现代农业，集约化、专业化、组织化和社会化水平稳步提高，建设现代农业园区231个、粮食生产功能区80万亩。

　　五年来，我们坚持促改革、扩开放、增合作，发展活力不断增强。全面深化改革，实施重点改革项目110个，商事制度、金融保险、新型城市化、社会事业、农业农村等领域改革加快推进，新增上市公司25家、“新三板”挂牌企业65家，巨灾保险稳步实施，民间投资占比达到48.1%。加大政府改革力度，推行政府法律顾问制度，支持仲裁机构建设，调整完善大榭开发区管理体制，行政审批事项大幅减少，非行政许可审批事项全面取消。扎实开展党的群众路线教育实践活动和“三严三实”专题教育，认真接受人大依法监督、政协民主监督、司法监督和社会监督、舆论监督，办理代表建议2951件、政协提案2915件，制定和修改政府规章43件。实施城市国际化行动纲要，新增国家级开发区（功能区）3个、友好城市26个，成为“一带一路”倡议支点城市。开放型经济提质增效，外贸出口总额位居全国第八位，跨境电子商务进出口总额位居全国试点城市前列，完成境外投资83亿美元。以“宁波周”活动为载体，深化与国内外城市合作，实际利用外资172亿美元、引进内资3324亿元，浙商甬商项目实际到位资金2165亿元。对口支援帮扶和“山海协作”取得新成效。

　　五年来，我们坚持抓统筹、促协调、优环境，城乡面貌加快改善。推进新型城市化，拉开现代都市框架，推动城市基础设施和公共服务向农村延伸，常住人口城市化率从68.6%提高到71.1%，城乡统筹发展水平居全国前列。加快中心城区建设，构筑“两心一轴、三江六岸”核心景观体系，东部新城、南部商务区基本建成，镇海新城、“两江北岸”建设稳步推进，东钱湖成为国家级旅游度假区。加强城市规划建设和精细化管理，打通“断头路”80条，整治主干道40条、背街小巷274条。区域统筹力度加大，南北两翼发展焕发新活力，“千人计划”产业园、宁南贸易物流区、南部滨海新区、象保合作区等特色平台加快建设，卫星城市地区生产总值年均增长12.8%。幸福美丽新家园建设成效明显，建成全面小康村613个、中心村101个、特色村85个，完成农房“两改”3000万平方米、21.9万户，农村发展面貌和农民生活水平逐步改善。城乡基础设施建设步伐加快，杭甬客专、绕城高速、穿山疏港高速、象山港大桥建成投用，轨道交通形成“十字型”运营骨架，城乡客运一体化率达到95%，能源、通信、污水、垃圾处理设施进一步完善。加强生态文明建设，实施循环经济、低碳城市试点，“五水共治”“三改一拆”“四边三化”稳步推进，消灭垃圾河282公里、治理黑臭河647公里，“三改”、拆违分别完成5841万平方米和5345万平方米，淘汰落后产能企业569家、黄标车11万辆，单位生产总值二氧化碳排放量累计下降20%，主要污染物减排任务全面完成。

　　五年来，我们坚持保基本、办实事、防风险，民生福祉全面提升。加大民生支出力度，公共财政用于民生投入年均增长17.2%。城乡居民收入稳步提高，年均分别增长10.5%和11.7%，收入比缩小到1.81∶1。推进国家级创业型城市建设，培育创业主体49.3万家，城镇新增就业81万人。社会保障逐步健全，基本养老、基本医疗保险参保人数分别达到403万和382万，最低工资标准、企业退休人员基本养老金、城乡居民基础养老金标准分别提高69%、46%和162%。养老服务体系基本建成，每千名老人拥有养老床位数达到36张。实现扶残助残爱心城区创建全覆盖。城乡居民人均住房面积分别达到40.3平方米和49.3平方米。打造文化强市，文化广场建成投用，奥体中心加快建设，中国大运河（宁波段）申遗成功，基本形成“城市15分钟、农村30分钟”文化服务圈。繁荣发展社会事业，改扩建中小学（幼儿园）510所，教育公平水平居副省级城市首位，每千人医生数达到3.7人。深化平安宁波建设，公共安全、应急管理、防灾减灾和“12345政务热线”整合扎实推进，社会治理法治化进程加快，食品药品安全监管得到加强，安全生产形势持续稳定好转。防范处置企业“两链”风险，加强房地产市场调控，金融生态环境、政府性债务结构持续改善。哲学社会科学、档案史志事业取得新进步。国家安全、国防动员和双拥工作不断深化，老龄、计生、工青妇、关心下一代、慈善、红十字事业稳步发展，民族、宗教、外事、侨务、港澳台等工作进一步加强。

　　各位代表！在国内外形势严峻复杂、困难挑战增多、自然灾害多发的情况下，过去五年的发展成绩来之不易。这是党中央国务院、省委省政府和市委科学决策、正确领导的结果，是市人大、市政协有效监督、大力支持的结果，也是全市上下团结奋斗、顽强拼搏的结果。在这里，我代表市人民政府，向全市人民，向市人大代表、市政协委员，向离退休老干部，向各民主党派、工商联、人民团体和社会各界人士，向驻甬人民解放军和武警部队官兵，向在甬部省属机构，向“宁波帮”和帮宁波人士，向所有关心支持宁波发展的海内外朋友们，表示衷心的感谢！

　　各位代表！面对新形势、适应新常态、实现新发展，我们也清醒地看到发展中还存在不少问题。主要有：经济增长动力不够强，新兴产业、现代服务业发展相对滞后，实体经济运行比较困难，创新驱动、改革推动和开放带动需要进一步加强；中心城区集聚辐射能力、城乡融合发展水平有待提高，宁波都市区建设需要进一步加快；资源约束趋紧，环境承载力下降，人口老龄化压力增大，经济社会领域潜在风险较多，基本公共服务、社会治理和民生保障力度需要进一步加大。同时，政府工作水平有待提升，一些工作人员的看齐意识、法治意识、改革意识和担当意识不够强，懒政怠政、消极腐败现象仍然存在，法治政府、创新政府、廉洁政府、服务型政府建设任重道远。对此，我们将坚持目标导向和问题导向，采取更加有力的措施，认真加以解决。

　　二、“十三五”发展的主要目标和重点任务

　　“十三五”发展，既面临大有作为的重大战略机遇期，也面临诸多矛盾相互叠加的严峻挑战。根据市委关于制定“十三五”规划的建议，我们制定了“十三五”规划纲要（草案），提请大会审议。

　　今后五年，我市发展的主要目标是：高水平全面建成小康社会，为全面建成现代化国际港口城市打下坚实基础。地区生产总值年均增长7.5%，一般公共预算收入年均增长7.5%，居民收入提高与经济增长同步，力争提前实现“四翻番”，使综合实力更强劲、城乡区域更协调、生态环境更优美、人民生活更幸福、治理体系更完善。

　　围绕上述目标，我们将坚持以“五大发展理念”为引领，按照跻身全国大城市第一方队和建设中国特色社会主义“四好示范区”的要求，着力建设创新型城市，着力打造港口经济圈，着力构建宁波都市区，着力提升国际化水平，加快构筑“一圈三中心”，加快形成引领经济发展新常态的体制机制，在厚植优势中增强发展动力，在补齐短板中提升发展整体性。

　　一是打造创新大平台，进入全国创新型城市和人才强市行列。加快新材料科技城、国际海洋生态科技城、宁波杭州湾新区建设，支持中科院宁波材料所、中国兵科院宁波分院等平台做大做强，争创国家自主创新示范区。规划建设一批特色小镇，打造集聚创新要素、发展特色产业、“产城人文”有机融合的功能平台。培育众创、众包、众扶、众筹空间，推动大学生留学生创业园、“千人计划”产业园等平台提升发展。强化企业创新主体地位，推进产学研协同创新，壮大创新型初创企业和高成长企业，形成一批具有国际竞争力的创新型领军企业。到2020年，高新技术企业达到2500家，众创空间和创客服务中心达到100家，高新技术产业增加值占规上工业增加值比重提高到40%，研究与试验发展经费支出占地区生产总值比重达到3%。创新财政科技资金分配机制，发展科技服务业，深化科技成果使用处置和收益权管理改革，不断优化创业创新生态体系。加强创业创新人才培养引进，推进国际教育合作，创新职教人才培养模式，完善人才评价激励机制，加快建设人才生态最优市。

　　二是构建开放大格局，打造港口经济圈、经贸合作交流中心和港航物流服务中心。深度融入“一带一路”、长江经济带战略，加快构建义甬舟开放大通道，规划建设梅山新区，建设跨境电子商务综合试验区，争设综合保税区、自由贸易港区和国家临空经济示范区。打造多式联运国际枢纽，共同建设江海联运服务中心，推进甬金铁路、甬舟铁路、沪嘉甬跨海通道、杭甬高速复线等项目，加快建设“海甬欧”贸易物流通道和宁波—华东地区集装箱海铁联运示范通道。到2020年，宁波舟山港集装箱吞吐量达到2600万标箱以上。完善“海上丝路指数”体系，提升航运金融、航运保险、航运人才、船舶交易的服务水平，打造国际海事服务基地，建成全国有影响力的多商品定价和交易中心。加快浙江海洋经济核心示范区建设，推进海港、海湾、海岛联动发展，打造海洋特色新兴产业基地和科技兴海创新基地。培育国际竞争合作新优势，打造国际贸易总部基地，建设国家进口贸易促进创新示范区，促进利用外资和“走出去”扩量提质。优化开放合作制度环境，扩大国际人文交流合作，提高城市开放度和国际影响力。到2020年，外贸进出口总额超过1200亿美元。

　　三是促进产业大转型，建设制造业创新中心和经济强市。推进信息化与工业化、制造业与服务业融合发展，做大做强新材料、高端装备、新一代信息技术、港航物流和生命健康产业，提升发展绿色石化、智能家电、时尚纺织服装等优势制造业，在文化创意、金融、旅游、海洋高技术、新能源汽车、通航产业等领域形成一批新增长点。提升制造业核心竞争力，推进“四换三名”工程，争创“中国制造2025”试点城市，打造标准强市、质量强市、品牌强市和制造强市，建设一批新型工业化示范基地，促进国际产业合作园、产业集聚区和开发区提质增效发展。深化国家服务业综合改革试点，做精做强贸易物流、现代金融、创意设计、信息服务等生产性服务业，推动旅游休闲、健康养老、教育文化体育等生活性服务业向精细化、品质化转变，建设一批月光经济夜市街区，打造国家电子商务示范城市和国内一流休闲旅游目的地。着力构建金融生态示范区，争创国家级保险创新综合试验区，加快全国普惠金融综合示范区建设。到2020年，服务业增加值比重提高到50%。实施“互联网+”行动计划，构建城市大数据中心，完善信息基础设施，推动信息经济蓬勃发展。建设国家现代农业示范区，推进粮食蔬菜生产功能区、主导产业集聚区和绿色都市农业示范区建设，壮大现代种业、远洋捕捞业、农产品加工业、休闲农业和生态循环农业。

　　四是推动功能大提升，形成更具集聚辐射能力的宁波都市区。加强与舟山协调发展，扩大与台州、绍兴、嘉兴等城市的合作，推动创业创新互促共进、交通设施互联互通、公共服务共建共享、生态环境协同共保。加快新型城市化，实施主体功能区战略，优化城市功能结构和市域城镇体系，增强中心城区集聚辐射功能，促进北翼地区融合发展，推动南翼地区统筹发展，加快卫星城市和中心镇创新发展，推进四明山区域生态发展。到2020年，常住人口城市化率达到74%。健全现代化基础设施，推进东部新城、姚江新城、空港新区建设，建成栎社国际机场三期扩建工程和轨道交通1号线、2号线、4号线、3号线一期，推进杭甬城际铁路项目和轨道交通5号线一期、6号线，组建运营宁波航空公司，加快中心城区至县（市）城际铁路建设，完善快速路网和跨江通道，创建国家公交都市，基本建成“两江同治6+1”工程。加快城乡统筹发展，深化户籍制度改革和农村产权制度改革，推进城乡基础设施一体化和基本公共服务均等化，加快智慧交通、智慧城管、智慧民生发展，完善综合行政执法体制，争创全国城乡统筹发展示范城市。提升城市文化品位，弘扬“宁波精神”，完善公共文化服务体系，创建全域化更高水平的文明之城，基本建成特色鲜明的文化强市。推进国防教育、国防动员、人民防空和双拥优抚安置工作，促进军民融合深度发展，争创全国双拥模范城“七连冠”。

　　五是抓好环境大优化，创建全国生态文明先行示范区和投资环境最优城市。优化生态功能区布局，强化生态带管理和生态环境治理，培育生态经济和低碳循环产业，倡导绿色生活方式，全面节约高效利用资源，完善生态文明制度体系，创建国家级生态园林城市。深入实施“提升城乡品质，建设美丽宁波”行动计划，加快建设品质城区、美丽县城、特色城镇和富美农村，基本实现县县无违建。到2020年，主要污染物排放总量大幅减少，黑臭河和地表劣V类水质断面全面消除，PM2.5浓度明显下降，生态安全屏障基本形成，使宁波的天更蓝、水更清、地更净、景更美。深化以“四张清单一张网”为重点的政府自身改革，以要素配置市场化为重点的经济体制改革，以基本公共服务均等化为重点的社会体制改革，以创新基层社会治理为重点的乡镇（街道）行政体制改革，在全省率先基本建成法治政府，构建法治化、国际化、便利化水平更高的国际营商环境。

　　六是确保民生大改善，构建品质更高的民生幸福之城。坚持民生优先，创新供给方式，促进基本公共服务总量扩大、质量提高、均衡发展，不断提升人民群众的获得感。深化国家级创业型城市建设，推行终身职业技能培训制度，加强高校毕业生、农业转移人口、就业困难人员等重点人群的创业就业服务。促进城乡居民持续增收，力争农村居民收入增长高于城镇居民收入增长、低收入农户收入增长高于农村居民收入增长。建成覆盖城乡、公平普惠的社会保障体系，加快改善困难群众居住条件，健全以低保为基础、“救急难”为重点的社会救助体系。到2020年，户籍人口基本养老保险、基本医疗保险覆盖率达到95%。率先实现教育现代化，构建公益普惠学前教育公共服务体系，促进义务教育优质均衡，推动基础教育水平提升，引进和打造若干所高水平大学和特色学院，建成国家现代职业教育开放示范区。推进健康宁波建设，深化医疗卫生事业改革发展，健全覆盖城乡的基本医疗卫生制度，完善分级诊疗体系，扶持中医药事业发展，提高居民健康水平。强化人口综合服务管理，积极应对人口老龄化。加强和创新社会治理，完善立体化社会治安防控体系，建成公共法律服务体系，实施最严格的食品药品质量追溯制度，不断提升公共安全保障水平。

　　各位代表，今后五年的目标任务十分繁重。我们坚信，经过全市人民的共同努力，“十三五”发展的宏伟蓝图一定能够实现！

　　三、2016年主要工作

　　2016年是“十三五”的开局之年，也是实现市第十二次党代会目标、完成经济社会转型发展三年行动计划的最后一年。当前，国内外经济形势错综复杂，不确定因素很多，下行压力依然较大。综合分析各方面因素，建议2016年经济社会发展的主要预期目标为：地区生产总值增长7.5%，一般公共预算收入增长7.5%，宁波舟山港集装箱吞吐量增长6%；城乡居民人均可支配收入分别增长7.5%左右和7.5%以上，城镇新增就业15万人，居民消费价格指数103%左右；节能减排降碳指标完成省定任务。

　　2016年工作的总体要求是：全面贯彻党的十八大和十八届三中、四中、五中全会精神，认真落实习近平总书记系列重要讲话精神，按照“五位一体”总体布局和“四个全面”战略布局的要求，以“五大发展理念”为引领，以“八八战略”为总纲，深入实施“六个加快”和“双驱动四治理”战略决策，扎实推进经济社会转型发展三年行动计划，适应和引领经济发展新常态，坚持改革开放，坚持稳中求进，千方百计扬优势，全力以赴补短板，坚持不懈增活力，统筹兼顾促均衡，努力保持经济社会平稳健康发展，实现“十三五”发展良好开局。

　　做好2016年工作，需要我们突出重点、开拓进取，以新作为谋新发展。围绕扬优势，我们将抢抓机遇、深化改革，不断放大港口优势、提升开放优势、增强制造业优势、扩大消费优势，提高发展的质量效益；围绕补短板，我们将找准问题、聚焦难点，着力强化创新驱动、扩大有效投资、壮大服务经济、加强环境治理、防范化解风险，增强发展的整体性；围绕增活力，我们将创新举措、改进服务，大力促进企业降低成本、消化库存和增强实力，提升发展的动能；围绕促均衡，我们将统筹兼顾、注重共享，扎实推进城乡品质、公共服务和民生保障持续优化，增进发展的协调性。

　　在具体工作中，把加强供给侧结构性改革放在突出位置，着力抓好以下五个方面的工作：

　　（一）突出发展质量效益，提高创新发展水平

　　推进“三年行动计划”。全力抓好“五水共治”、现代产业培育、城市功能提升、民生福祉改善、发展基础强化“五大工程”建设，确保“三年行动计划”圆满收官。新开工重大项目186个，续建327个，建成153个，完成投资2190亿元。加强重大项目的谋划和储备，优化土地和资金保障，推动PPP试点项目落地实施。谋划编制新一轮“三年行动计划”。

　　强化创新驱动发展。实施“科技领航”和创业引领计划，培育一批创新型初创企业、高成长企业和领军企业。引导企业加强技术、产品、管理和商业模式创新，推动企业走出去并购技术和品牌，支持企业上市，鼓励企业到“新三板”、股权交易中心挂牌。引导在甬央企、大型民企发展贸易物流等业态。建设产学研战略联盟，加快技术研发与应用产品示范推广，强化关键共性技术、装备和标准的研究攻关。推进新材料科技城和国际海洋生态科技城建设，积极培育特色小镇和众创空间，扶持科研院所和产业技术研究院加快发展，争创国家自主创新示范区。开展国家科技和金融结合试点地区建设，争创国家科技成果转化行动试点城市，培育科技服务业和技术大市场，提高创新成果转化效率。加强知识产权管理服务，推进专利区域布局试点。研究与试验发展经费支出占地区生产总值比重达到2.45%。

　　增强产业竞争实力。积极争创“中国制造2025”试点城市。加快石墨烯、汽车电子、新能源汽车等新兴产业发展，深化“四换三名”工程，实施智能制造、绿色制造、“互联网+制造”示范工程，推动传统优势产业高端化、品牌化、国际化发展。规上工业增加值增长5.5%以上。推进建筑工业化。大力培育服务经济，加快现代金融、跨境电商、文化创意等产业发展，提升生活性服务业品质，推进宁南贸易物流区、电子商务“一城两区一中心”和电商经济创新园区建设。加快国家现代农业示范区和都市农业园区建设，完善现代农业经营和科技金融支撑体系，支持农村青年、高校毕业生、返乡人员领办合作社和家庭农场，培育发展农村电商等新业态，努力提高农业精细度、农村美丽度和农民富裕度。

　　落实企业减负稳增促调政策。完善企业减负机制，深入实施降低实体经济企业成本行动，切实降低制度性交易成本、税费负担、社保费率、财务成本、要素价格和物流成本，推动企业发展提质增效。积极破解小微企业发展空间、融资渠道、政策支持、公共服务等共性问题，推进小微企业产业园、标准厂房建设，支持小微企业创业创新发展。健全优胜劣汰机制，稳妥处置“僵尸企业”，鼓励企业兼并重组。以满足新落户居民需求和推进棚改货币购房为重点，管控土地供应，打通供需通道，推动房地产业平稳健康发展。

　　有效扩大“三大需求”。加大有效投资，优化投资结构，突出基础设施、新兴产业、生态环保和民生领域投资，固定资产投资增长10%以上。坚持“优出优进”，推动跨境电商、服务外包和名特优产品出口较快增长，积极扩大进口，力争外贸出口增长2%。培育新的消费热点，促进健康、养老、信息、旅游、文化、体育等新型消费，做大做强月光经济、节庆经济，提升市民“菜篮子”品质。完善商贸流通网络，实施宽带网络提速降费行动，强化消费维权法律保障。优化消费市场环境，加快商品交易市场提升和放心市场建设。社会消费品零售总额增长10%。

　　创新发展体制机制。深化“四张清单一张网”建设，加快行政审批制度改革，健全招投标监管协调机制，推进综合行政执法、税收征管和行政复议改革，全面完成新一轮公务用车改革。完善现代市场体系，实行差别化资源要素定价机制，推进公共信用信息平台建设，培育规范中介服务机构，完善政府和社会资本合作模式，健全质量评价指标体系。推进亩产倍增计划和建设用地总量与强度“双控”行动，强化城乡建设用地规模和开发强度的刚性约束。集聚发展总部型、特色型金融机构，启动运营股权交易中心，加快发展产业基金、PPP投资基金。强化经营性国有资产统一监管，做强做优国资运营平台。完善农村产权流转交易和宅基地有序流转机制。深入实施人才新政，优化人才服务机制，鼓励创办科技型中小企业。

　　（二）注重统筹协同联动，提高协调发展水平

　　健全基础设施。加快栎社国际机场三期扩建，组建宁波航空公司，推进甬舟铁路、沪嘉甬跨海通道、杭甬高速复线项目前期，力促甬金铁路开工建设。深化国家公交都市示范城市创建，建成东苑立交一期，推进轨道交通2号线二期、3号线一期和4号线建设，确保1号线二期通车运营。强化地下空间开发利用，推进“海绵城市”、地下综合管廊城市建设试点。建设智慧城市运营中心。

　　推进区域联动。深化“多规融合”，统筹抓好市区行政区划调整优化，构建统一衔接、功能互补、相互协调的空间规划体系。提升中心城区集聚辐射能力，加快空港新区、大榭穿鼻岛开发建设，完善科技创新、国际贸易、航运服务和总部经济功能，提高人才、科技、资本等要素集聚水平。推动余姚、慈溪和宁波杭州湾新区融合发展，延伸宁波杭州湾新区的产业链、创新链，加快姚北新城、胜陆公路建设，支持慈溪转型发展。促进奉化融入中心城区，推动宁海、象山跨越式发展，加快南部滨海新区开发建设，科学保护利用象山港区域。加强精准帮扶，推进四明山区域生态发展。

　　提升城乡品质。做精中心城区，实施核心景观、形象品质、文化特色、基础设施、民生服务、生态环境提升“六大行动”，加快中山路整治提升和“三江六岸”品质提升，推进夜景美化和塘河整治。做优美丽县城，抓好核心城区美化提质、县城与新区美丽连线、基础设施品质提升与交通畅行、特色文化传承与民俗文化彰显、公共服务保障与活力城市提升“五大行动”。打造美丽乡村升级版，实施农村环境卫生整治、生态环境建设、安居宜居美居、美丽乡村示范创建“四大行动”，加强村庄规划、农房设计和特色传统村落保护。推进“三改一拆”，深化无违建县（市）区、乡镇（街道）创建，加大城中村改造力度。加强城市精细化管理，完善村民自治，提升城乡治理水平。

　　协调推进“文化小康”。加强社会主义核心价值观建设，推进全域化更高水平文明之城创建。提升市民素质和社会文明程度，弘扬众创文化、诚信文化和爱心文化，打响“爱心宁波•尚德甬城”品牌。推进城市文化公园、农村文化礼堂和社区文化家园建设，抓好历史文化村落保护试点。深化文化体制改革，整合媒体、演艺、出版、影视等行业资源，推动媒体融合发展。培育文化产业，加快象山影视城发展，促进华强非遗博览园开园运营。举办首届宁波特色文化产业博览会，办好“东亚文化之都”系列活动，深化国际文化交流合作。实施全民健身工程，加快奥体中心建设，办好中日韩青少年运动会和宁波（梅山）国际马拉松赛。

　　（三）狠抓生态环境改善，提高绿色发展水平

　　加大环境保护治理力度。推进“五水共治”，严格落实“河长制”，加快“两江同治6+1”工程建设，抓好源头控制、截污纳管、达标排放、生态修复。强化工业废气、粉尘扬尘、建筑渣土和汽车尾气污染治理，加快燃煤电厂超低排放技术改造和高污染燃料锅炉改造淘汰，推进镇海电厂搬迁。深化“四边三化”“两路两侧”专项整治，推进矿山复绿和公路两侧青山白化整治。加强土壤污染监测和治理，抓好重金属污染防治。坚持依法治渔，加快渔场修复。加强生态功能区和生态带建设，完善生态环境动态监测网络，依法严惩环境违法行为，努力把绿水青山护得更美。

　　促进低碳循环发展。深化低碳城市试点，完善市场化碳减排机制，推行碳排放权交易。大力发展循环经济，加强废弃物资源化综合利用，扩大农村生活垃圾分类处理试点，城区生活垃圾无害化处理率达到100%。择优发展临港产业。大力推广绿色建筑。培育壮大节能环保产业，推进微电网和智能电网发展。

　　推动资源集约高效利用。探索资源环境要素交易制度。推广先进节能技术，实施节能改造项目500项，年节能50万吨标煤以上。推广合同能源管理，完善能耗强度总量“双控”责任制。加强用水总量和效率控制，提高工业用水重复使用率和污水处理厂再生水回用率。强化用地节地责任考核，大力清理闲置用地，开发利用低丘缓坡荒滩，复垦利用工矿废弃地，加强城镇低效地再开发，加大永久基本农田保护力度，进一步提高土地集约利用水平。

　　（四）坚持互利共赢合作，提高开放发展水平

　　推进港口经济圈建设。加快构建多式联运国际枢纽，完善沿海、长江内支线和近洋支线布局，推进梅山保税港区集装箱码头建设。共同建设义甬舟开放大通道和江海联运服务中心，加快沿海沿江中转网络和铁矿石分拨中心建设。健全宁波—华东地区集装箱海铁联运示范通道功能，拓展“海甬欧”货源，力争海铁联运集装箱量较快增长。完善港航服务体系，培育大型港口物流集团，加快临港物流产业发展，建立“海上丝路指数”平台。推进“三互”口岸大通关建设，全面完成国际卫生港创建。办好第二届中国—中东欧国家投资贸易博览会、中国航海日活动。

　　提升发展开放型经济。加强外贸自主品牌建设，培育本土外贸综合服务企业，推进跨境电子商务综合试验区建设，争创全国贸易便利化综合试验区，争取国家市场采购贸易、口岸通关便利化、跨境人民币结算和汽车平行进口试点，鼓励先进技术设备、关键零部件和资源性产品进口。扩大服务贸易规模，推进服务外包园区建设。优化利用外资环境，引进智能装备、海洋装备等优质大项目，实际利用外资45亿美元。积极培育“走出去”主体，加快建设境外投资创业基地，争创国家级境外经贸合作区。

　　完善国际化合作载体。积极谋划梅山新区建设，推进综合保税区、自由贸易港区申报。整合提升重点开发区域，加快中意宁波生态园、北欧工业园、中东欧工业园和象保合作区建设，促进特色园区国际化、高端化、品牌化发展。深化与中东欧国家城市的合作，支持企业到中东欧国家建设经贸产业合作园区。加强国际人文交流合作，大力引进多边国际会展、活动和赛事。充分发挥“宁波帮”和帮宁波人士作用，进一步密切与国际友好城市的交流合作。

　　深化区域交流合作。精心办好“宁波周”活动，深度融入长三角一体化发展，加强与国内中心城市和重点区域的交流合作，扩大投资贸易往来，拓展港口腹地。加大内资引进力度，促进浙商甬商回归。推进“活力浙东”建设，共建浙东经济合作区。深化“山海协作”和对口支援帮扶，推进山海协作产业园建设，提高援疆、援藏、援黔、援青等工作成效。

　　（五）优化公共服务供给，提高共享发展水平

　　拓宽增收通道。完善收入分配制度和劳动报酬增长机制，多渠道促进居民增收，加大对低收入群体增收的帮扶力度。实施就业优先战略和创业带动就业政策，加强高校毕业生就业服务和创业指导，培养新型职业农民，强化就业困难人员帮扶。完善人才和职业培训体系，实施“技能宁波”行动计划，建设世界技能大赛集训和全国高技能人才培养示范基地。

　　完善社保体系。深化医保改革，适度降低市区职工医保缴费费率。完善大病保险制度，实现职工和城乡居民大病保险政策体系、待遇范围、享受办法“三统一”。稳步推进机关事业单位养老保险制度改革。完善社会救助体系，扩大低保救助覆盖面，促进慈善事业发展。加强对特殊人群、特殊困难的精准帮扶。健全养老服务体系，完善医养结合模式，探索长期护理保险制度。推进公租房和廉租房并轨运行，强化房屋使用安全管理。加快以成片危旧住宅区为重点的棚户区改造，提高货币化安置率，新启动项目70个、面积500万平方米。

　　推进教育现代化。推动学前教育公益普惠、义务教育优质均衡、高中教育特色多样。深化中小学课程改革，加快转变育人模式。建设高水平大学和特色学院，推进与浙江大学的战略合作。加快培养高端技术技能型人才，深化国家职业教育与产业协同创新试验区、国家现代职业教育开放示范区建设。启动宁波供应链创新学院项目，推进教育国际合作与交流综合改革试验区建设。完善全民终身教育体系，提升特殊教育发展水平，支持民办教育发展。

　　建设健康宁波。深化医药卫生体制改革，推进李惠利东部医院综合改革试点，加快药品耗材采购和定价机制改革。建设区域医联体，加快优质医疗资源“双下沉、两提升”，构建分级诊疗体系，推进家庭医生制签约服务，让城乡居民享受更优质、更便捷的医疗服务。实施医教研一体化，推进宁波医学中心建设，启动市第一医院异地建设项目前期和市康复医院扩建项目。推进云医院建设，开展健康大数据应用试点。落实一对夫妇可生育两个孩子政策。做好国家卫生城市复查迎检工作。

　　提升群众安全感。完善基层社会治理“八大体系”，推进“网格化管理、组团式服务”，加快公共法律服务体系建设。推进信访制度改革，畅通群众诉求渠道，依法处理和多元化解社会矛盾。健全立体化社会治安防控体系，严密防范和严厉打击违法犯罪活动，深化出租房专项整治。创新流动人口服务管理。推进应急指挥平台和防灾减灾救灾体系建设，完善突发事件应对机制，促进巨灾保险更加惠民。强化安全生产主体责任，夯实安全生产基层基础。加强食品药品安全治理，建设国家食品安全示范城市。依法打击非法金融活动和恶意逃废债行为，推进不良资产化解处置，确保不发生区域性金融风险。支持国防建设和军队改革，形成军民融合深度发展新格局。

　　各位代表！2016年我们将继续加大力度，办好一批关注度高、惠及面广、当年见成效的民生实事，让广大人民群众共享更多更好的发展成果。

　　各位代表！新时期、新目标、新任务，对提高政府执行力和公信力提出了新的要求。我们将扎实推进法治政府建设，增强法治思维，科学民主决策，完善依法行政制度体系，严格规范公正文明执法，自觉接受人大依法监督、政协民主监督和司法监督，主动接受社会监督和舆论监督，切实把行政行为纳入法治的轨道。我们将扎实推进创新政府建设，加快政府职能转变，强化供给侧结构性改革和制度创新，积极运用互联网创新治理方式，完善支持科技创新、产业创新的体制机制，搭建创业创新、便民利民的服务平台，健全减轻企业负担、提高公共财政绩效的政策体系，推出激发市场活力、提升监管效率的工作举措，努力提供高效公共服务和优质公共产品。我们将扎实推进廉洁政府建设，深入践行“三严三实”，坚持不懈纠正“四风”，严格落实全面从严治党主体责任，严格规范“三重一大”集体决策行为，严格加强政府内部层级监督、专门监督和审计监督，以良好的政风谋发展、促改革、保稳定。我们将扎实推进服务型政府建设，深化政务公开，简化办事环节，加强统筹协调，提高服务质量，坚决整肃懒政怠政行为，坚决消除不作为和“中梗阻”现象，打通“最后一公里”，把政策措施落实到底，把公共服务送到基层，切实提高政府服务水平和群众满意度。

　　各位代表！干在实处永无止境，走在前列要谋新篇。让我们更加紧密地团结在以习近平同志为总书记的党中央周围，在市委的坚强领导下，振奋精神、真抓实干，为实现“十三五”发展良好开局，为高水平全面建成小康社会而努力奋斗！
